# Metallic Nanoparticles: General Research Approaches to Immunological Characterization

**DOI:** 10.3390/nano8100753

**Published:** 2018-09-22

**Authors:** Francesca Gatto, Giuseppe Bardi

**Affiliations:** Istituto Italiano di Tecnologia, Nanobiointeractions & Nanodiagnostics, Via Morego 30, 16163 Genova, Italy

**Keywords:** nanoparticles, immunology, cellular models, animal models, inflammation

## Abstract

Our immunity is guaranteed by a complex system that includes specialized cells and active molecules working in a spatially and temporally coordinated manner. Interaction of nanomaterials with the immune system and their potential immunotoxicity are key aspects for an exhaustive biological characterization. Several assays can be used to unravel the immunological features of nanoparticles, each one giving information on specific pathways leading to immune activation or immune suppression. Size, shape, and surface chemistry determine the surrounding corona, mainly formed by soluble proteins, hence, the biological identity of nanoparticles released in cell culture conditions or in a living organism. Here, we review the main laboratory characterization steps and immunological approaches that can be used to understand and predict the responses of the immune system to frequently utilized metallic or metal-containing nanoparticles, in view of their potential uses in diagnostics and selected therapeutic treatments.

## 1. Introduction

Nanoparticles (NPs) are extremely attractive for several biomedical applications, due to their capability to interact with molecular or cellular processes and the possibility to influence their functions [1,2,3,4,5]. Specifically functionalized NPs have the potential to overcome some limits of many traditional therapeutics, such as poor water solubility or lack of target specificity [6]. Metal-based NPs possess unique physicochemical properties, offering many advantages and potential usages [7,8] (Table 1). For example, the peculiar optical properties of plasmon-resonant noble metals (i.e., Au, Ag, Pt, and Pd) and luminescent semiconductor nanocrystals (quantum dots (QDs)) make them useful as markers for biological systems imaging [9,10,11,12,13,14,15,16,17,18,19,20,21]. Gold nanoparticles (Au NPs) find a wide spread use in biological applications, such as cancer therapy [22], cell labelling [23], drug delivery [24], and diagnostics [25]. Aluminium (Al) and iron oxide (Fe_2_O_3_) NPs have been proposed as drug delivery systems [26,27,28]. Fe_2_O_3_ can be used exploiting their magnetic features to drive loaded NPs to specific target tissues by applied magnetic fields. Furthermore, other metal-based NPs have been deeply investigated as candidates for novel antimicrobial applications. Silver NPs are, indeed, widely used in medicine, and in common household products as additives with antimicrobial activity against more than 650 different types of disease-causing organisms, including viruses [29,30,31,32,33,34,35,36,37,38]. Along with Ag, TiO_2_ and Cu NPs also show strong antibacterial activity [39,40]. Different applications of Ag NPs have been explored, such as cancer therapy and wound healing, spreading their exploitation in several fields of biomedicine [41,42,43,44]. Interestingly, the catalytic properties of metal NPs could suggest their potential use in oxidative stress-based disease, as indicated by Reactive Oxygen Species (ROS)-scavenging activity of Pt NPs [45,46]. However, the same properties may lead to unexpected outcomes when NPs interact with biological tissues. Indeed, the biological effects of materials at the nanoscale cannot be anticipated by the knowledge of the same materials in the bulk form [47,48,49]. The understanding of the cause–effect relationship between the nanomaterial properties and their interference with biological processes would enable the prediction of unforeseen hazards and the synthesis of “safe-by-design” nanomaterials [50]. The safety assessment of NPs must include the consideration of three major topics: an NP’s physicochemical characteristics, fate (biological or environmental), and (re)activity [51].

The compatibility with the immune system (IS) represents a crucial issue to comprehend an engineered NP’s biological fate, and to approach feasible nanomedical applications [52]. The IS is organized in a complex defense structure, tailored by evolutionary processes. It has a protective role against foreign bodies, toxic and pathogenic entities considered “non-self” or dangerous, which are safely confined or eliminated [53]. Among the mechanisms aimed at entrapping non-self bodies, the major importance of the so-called “opsonization” phenomenon is recognized. This event involves specific proteins, like antibodies, the complement enzyme-protein system, or membrane receptors with the capability of firmly bind other structures, such as lipoproteins or sugars [54].

Specialized immune cells expressing many of these receptors belong to the innate immune system, the fast responding and non-specific defense against any invading threats for the organism. These cells are characterized by two main activities: phagocyting and/or killing pathogens. In particular, neutrophils, macrophages, and dendritic cells perform phagocytosis driven by those receptors whose cognate agonists “label” the microorganism. On the contrary, NK cells and granulocytes release cytotoxic substances once stimulated or in contact with warning signals.

Two major classes of key membrane proteins mediating immune responses are the antibody-binding fragment crystallizable region receptors (FcRs) and the membrane associated pattern-recognition receptors (PRRs), binding to a variety of conserved pathogen-associated molecular patterns (PAMPs), which distinguish the foreign bodies, such as viruses or bacteria [53]. A set of soluble proteins binding microorganisms is the complement system. Its name reminds the “complementary role” to antibodies, participating together in the several mechanisms to remove intruders and damaged cells [55]. Such enzyme-activated pathway of opsonization is gaining evidence as one of the most important biological effects of nanoparticle delivery [56].

NPs released in the biological milieu adsorb several of the described molecules on their surface, facilitating the interception and internalization by immune cells. The set of molecules adsorbed onto the NPs is generally called “corona”. Most of them are recognized by cell surface receptors which are able to uptake the opsonized particle by different pathways, often depending on the NP–corona complex size [57]. Hence, engineered man-made materials could be considered as non-biological danger signals for the immune system and may, either directly or indirectly, activate phagocytosis. Hence, it is postulated that immune cells could sense nanomaterials, which are designated as nanoparticle-associated molecular patterns (NAMPs) [58]. This may be accomplished via surface-adsorbed biomolecules, or it could result from the recognition of specific structures of the nanomaterial per se.

NPs or NP–protein aggregates may accumulate in the subcellular compartments, interfering with the physiological immune functions and triggering inflammatory signaling [59]. For example, in macrophages, different metallic NPs or non-degradable particles can accumulate and activate cytosolic inflammasome complexes [60] which regulate the proteolytic enzyme caspase-1 [61]. Caspase-1 induces the maturation of proinflammatory cytokines, in turn, triggering inflammation [62]. On the other hand, the mechanism of NP escape from the cell is not so clear [63], although evidence of the exocytosis mechanisms has been described [64].

Physicochemical characteristics, like size, shape, and surface chemistry of the particles, regulate most of the aspects related to the bio-interaction [65]. Moreover, additional materials used to increase metal NP solubility or as biocompatible coatings, may also change NP interface with the biological environment, including their immunological profile [66]. Although significant improvements in nanotechnology have occurred in many years of research, precise relationships between NP physicochemical features and their biological outcomes still represents an arduous task to be elucidated. Major challenges in immunological studies require meticulous characterization and the choice of suitable experimental models. The intrinsic properties of nanoscale materials often complicate the resolution of several assays and instruments currently used in biochemistry, leading to many inconclusive and contradictory data on NP-induced responses [67].

The focus of this review is to outline the main characterization steps and the current immunological approaches to understand IS interaction and its inflammatory responses to commonly used metal-based NPs.

## 2. In Vitro

*In vitro* studies are a fast approach to evaluate NPs’ reactivity and induced toxicity, as well as their cellular uptake and inflammatory potential [68]. In order to make a meaningful comparison among different experimental results across different studies, it is imperative to carry out a precise nanomaterial characterization, along with standardization of the experimental protocols.

Currently used *in vitro* methodologies require high accuracy in the assessment of the potential NP-induced immunotoxicity, as several issues may compromise the results. These include the choice of the appropriate cell culture model; NP dose and dose metric; relevant and suitable positive and negative controls; the assay format; the selected endpoint; the NPs’ interference with the assays; and, last but not least, the understanding of assay predictability for the corresponding immunotoxicity *in vivo*. Sometimes, it is undervalued the importance of reliable cell culture models mimicking the actual human immune systems. Different immune cells perform different tasks, and they have diverse protein expression profiles and uptake mechanisms. In addition, frequently used *in vitro* assays involve murine or human cell lines (tumor derived or “artificially” transformed) demonstrating advantageous robustness and facile growing conditions, but may not reflect the natural conditions of the primary cells.

Although the use of cell lines in monoculture systems is recommended for the first stage of safety evaluation, the relevance of specific, more advanced *in vitro* models (i.e, co-cultures or three-dimensional (3D) models) has been highlighted. These models would be preferable for assessing NP interactions and cellular effects, in order to overcome the lack of phenotypic details, physiological functions, and complex cell crosstalk of the traditional monocellular type cultures [69]. For instance, to study gastrointestinal NP exposure, a feasible *in vitro* model could be the simulation of the intestinal barrier by enriching the epithelial cell layer with macrophages, in order to comprehend their functional crosstalk in the generation of inflammatory responses [70]. The cellular model also influences the exposure duration and the relative endpoint assessed, posing limitation of sub- and chronic exposure studies. Major restrictions depend on the fast cell division of most cell lines or the possible de-differentiation during prolonged culture time. In this regard, *in vitro* systems might not be suitable to evaluate any potential dysregulation of immune system long-term responses [71].

### 2.1. NP Dose

Metal NP dispersion in liquid media represents a critical condition to assure the expected dose to be delivered. The NP suspension preparation must be optimized to avoid aggregation, dissolution, or detachment of functional ligands that could affect the final testing concentration. The cell culture media also alters NP characteristics, mainly because of the presence of serum proteins forming the protein corona and affecting NP agglomeration, sedimentation, as well as their overall biological identity. The serum source (i.e., bovine, calf, horse, human), its treatment and manipulation (i.e., heat-inactivation), as well as the final concentration in culture (e.g., 10% or 1%) used can affect NP interaction with cells, modulating particle uptake and toxicity. Serum interference could be avoided by performing *in vitro* experiments in serum-free conditions, but serum-component deprivation is an unrealistic condition, strongly affecting cell behavior and viability.

The choice of an appropriate dose of exposure could compromise immunotoxicity of a certain NP. Albeit *in vitro*, the assessment of potential immune response should be based on a realistic human exposure to NPs [72,73,74]. Often, unrealistic high concentrations are used for the determination of NP effects. An obstacle is represented by the evaluation of the discrepancy between the administered and the deposited NP amount in the experimental dishes. Dosimetry curves for metal NPs could be defined by elemental analysis of cells treated with metal NPs by inductively coupled plasma atomic emission spectroscopy (ICP-AES) or inductively coupled plasma mass spectrometry (ICP-MS), which are precise methods to evaluate plausible NP concentrations targeting the cells. Unfortunately, these methods do not discriminate between the number of NPs stuck on the cell surface and the ones inside the cell. Many cell washing steps at the end of metal NP administration are helpful, although the procedure of etching metal NPs from the surface would be suggested before NP quantification [75].

### 2.2. NP Interference

It is worth reminding that specific physicochemical properties of NPs, in particular the metal ones, can interfere with established tests originally developed for biological samples using conventional chemicals [76]. Due to their increased surface to volume ratio, NPs display an increased adsorption capability. The affinity to bind polypeptides may strongly influence the readout of protein concentration or their activity [77]. Due to their optical properties, metal-based NPs present in the reaction mixture or in cell culture medium compromise several assays based on light absorption or fluorescence detection [78,79,80]. The possible NP interferences with the optical reading (absorbance, luminescence, or fluorescence) could lead to false positive results. This imposes several controls, including the NP suspension alone, that may cause optical effects by itself. False results could also arise from peculiar catalytic properties of some metallic NPs.

A comprehensive and reliable understanding of NP effects on the immune cells can be achieved only using specific assays, but, despite global efforts, there are no universal established standards for the abovementioned areas [71,72,81].

### 2.3. Cytotoxicity

The impairment of cell viability is the first parameter to evaluate the NP potential immune response. Many observed inflammatory outcomes are actually due to cell death and intracellular molecule release in the medium, emerging as consequences of cell or tissue damage.

Since several cell types are commercially available, cells belonging to the presumed target tissue of our nanomaterial should be preferred. Following this rationale, toxicity tests should probe the viability of the immune cells patrolling the specific tissue of interest. For example, NPs designed for the release of drugs in the bloodstream could be monitored for their potential dose-dependent cytotoxicity with endothelial cells and blood resident immune cells. Equally, brain-aimed NPs must be tested with neurons, glia, and microglia cells.

As a result of several cellular processes, the viability could be assessed considering different outcomes: detection of mitochondrial activity (MTT, MTS, WST colorimetric assays) [82], evaluation of necrotic and apoptotic processes (LDH release, annexin V/propidium iodide staining, caspase-3 detection) [83,84,85,86], and the assessment of lysosomal integrity (neutral red uptake) [87]. Complementary to NP impairment of cell viability, proliferation assays could give evidence about the NP-triggered effect on the normal cell cycle in actively replicating cells. On the contrary, interference with the differentiation process could also be considered to define the possible misleading regulation of cell maturation [88].

As previously mentioned, NP-induced cytotoxicity of fast-proliferating cells can be quite different from its toxic effect on primary cells with a physiological cell cycle. Thus, the use of cell lines is limited to the evaluation of validated biomarkers reflecting the actual response of normal human cells [89]. NPs could also induce an indirect effect by the adsorption of growth factors and nutrients from the cell culture medium [90], interfering with the readout. In toxicity tests using propidium iodide, false positive samples occur by the presence of this dye stuck on metal NP surface, increasing the uptake by viable cells, but finally counted as dead cells [91]. Another example of interference with metabolic tests is the NP interaction with the enzymatic substrate causing the depletion of the free form of the latter (i.e., MTT) and producing false negative results [92]. Metal ions have been clearly shown to interfere with the LDH assay [93,94], and several metal NPs may absorb light, quenching probe fluorescence in different tests [93,95,96,97]. The catalytic activity and the magnetic properties of some metal oxides may also cause erroneous signals in detection methods based on redox reactions [98,99,100,101,102]. For example, metal ions derived by the dissolution of the NPs interfere with the MTT reduction reaction [78].

Along with viability tests, the cellular stress response gives information regarding active cell reaction to exogenous materials. Reactive oxygen species (ROS) are generated by cells as byproducts of normal cellular activity, and are neutralized by cellular antioxidant defenses. If the ROS production exceeds the cell neutralizing capacity, an oxidative stress status occurs. To monitor this oxidative status, the two most common assays are the measurement of ROS generation and the glutathione (GSH) reduction. The generation of ROS is usually investigated using a fluorometric assay based on intracellular oxidation of 2’,7’-dichlorodihydrofluorescein diacetate (DCFH-DA) [103], while GSH is measured with assays based on the production of a fluorescent or colored dye upon reaction with GSH [104]. As many fluorescent probes, however, results using DCFH-DA suffer from the interference of metallic NPs, showing plasmon resonance in the visible spectrum and specific absorbance around 490 nm wavelength [105]. Furthermore, the catalytic properties of some metal particles must be taken into account since non-internalized deacetylated DCF may accumulate in the extracellular space, and could react with catalytically active substances outside the cells [106].

Intracellular Ca^2+^ release is another useful indicator of cell activation. Several cellular mechanisms are triggered by an increase in intracellular Ca^2+^ concentration, potentially initiating apoptotic signals or autophagy [107]. Since most of the Ca^2+^ probes are fluorescent, the same metal NP-induced optical interference could be found.

### 2.4. Inflammatory Potential

An exquisite parameter to assess cellular immune response is the production and release of inflammatory markers, like cytokines and chemokines. The evaluation of this type of response is commonly conducted using enzyme-linked immunosorbent assays (ELISA), that enables simple and accurate quantification of released markers by specific antibodies and enzymatic reactions.

Multiple-parameter analysis can also be performed using multiplex systems [88,108,109]. Cytokine release represents a key activity of immune cell to coordinate either inflammatory or anti-inflammatory responses. Although cytokine release is peculiar for each cell type determining following precise activation fates of the target cell (e.g., lymphocyte polarization in different sub-population depends on specific monocyte/macrophage cytokine released pattern), immune cell lines often show defined expression patterns commonly shared by several types of NP insults. Similar inflammatory responses obtained *in vitro* do not always mean that all NPs behave in the same way inducing the same response. Specific cell line-gene rearrangement may limit the response to similar expression patterns facing particles made of diverse materials, which probably behave differently in primary cells derived from the same tissue.

Along with these well-known markers, the expression of membrane receptors involved in the cellular immune response can give the researcher important information [88,108]. Membrane proteins, as well as lipids, denote the activity of cells in the different physiological or pathological conditions. Upregulation of CD11b (cluster of differentiation 11b), for instance, can provide an indication of macrophage and microglia activation [110]. Increased expression of a certain protein receptor prepares the cell to respond quickly to its cognate agonist. For example, upregulation of FcRs (immunoglobulin receptors) amplify the macrophage ability to engulf opsonized external bodies, including NPs. Flow cytometry and confocal microscopy are commonly performed to evaluate membrane protein expression. The first represents a suitable analytic method for non-adherent cells (e.g., monocytes) and allows precise and fast quantification of different labelled targets, contemporaneously expressed on the cells [111]. Confocal microscopy, otherwise, is recommended to observe fluorescently probed objects in adherent cells (e.g., macrophages) [112]. Both techniques exploit fluorescence and have been developed for biological samples. As described above for other optical detection techniques, it is suggested to carefully include the proper controls to avoid artifacts created by NPs. Non-specific interactions of protein-labelling antibodies with NPs, or even more likely, with proteins absorbed onto the NPs, could induce variations in the detected fluorescence invalidating the data.

Another key feature of inflammatory responses is the immune cell trafficking and localization in the insulted tissue. Immune cell trafficking in peripheral and lymphoid organs is mediated by specific chemoattractant cytokines (known as “chemokines”) and their cognate heptahelical transmembrane receptors [113]. The release of chemokines by inflamed tissues creates a signaling gradient, guiding leukocyte migration to the target site. Persistent metallic NPs endocytosed by immune cells could compromise their ability to reach the inflammation source, impairing their response to chemotactic stimuli. This occurrence is always underestimated. Nevertheless, useful information on the potential reduced, or increased, chemotaxis ability, can be easily tested *in vitro* by Boyden chambers or Transwell plate systems [114]. The eventuality of altered NP-induced cytoskeleton rearrangements may have important consequences on the kinetics of the immune response.

### 2.5. Colloidal Suspension Impurities

NP-induced immunological profile imposes the distinction of NP-mediated effects from those triggered by chemical and biological impurities that are not always completely removed during the NP synthesis. These impurities might be responsible for unwanted effects, as suggested by different studies [71,93,115]. Among the possible contaminants, the presence of endotoxins [116] can lead to false positive results [117]. Some nanomaterials, while not inflammatory themselves, are able to potentiate endotoxin-mediated inflammation [118,119,120]. Currently, the FDA-approved method to detect endotoxin is the limulus amoebocyte lysate (LAL) assay for *in vitro* tests.

While many types of NPs have been linked to certain types of immunotoxicity, there are no reference materials to be used to standardize immunotoxicity results. Usually, immunological studies are conducted using traditional controls, such as bacterial lipopolysaccharide (LPS) as an inducer of cytokine production [121].

## 3. General Considerations on *In Vivo* Investigation

The study of the whole organism provides information on the immune response together with systematic data on NP pharmacokinetics, tissue absorption, distribution, metabolism, accumulation, and excretion [122]. The immune reaction has a dynamic nature and a defined time sequence, turning in a rapid deactivation once the cause has been eliminated. When the defense response is abnormal, by duration or distribution, a pathological state occurs. In this context, the evaluation of a selected biomarker does not always discriminate between pathological inflammation and physiological reaction. Major attention should be paid to the detailed profile of response kinetics [71].

NPs could enter the body through six main routes: intravenous, oral, intraperitoneal, inhalation, dermal, or subcutaneous [123]. The exposure route has enormous importance in the potential response of the immune system. Different antibody molecules are generated and released in the different tissues, so conditioning the eventual opsonization of NPs and their sequestration by patrolling phagocytes [124]. For example, IgA antibodies dimers intercept NP released by an oral route, and may induce the activation of an IgA-mediated response. On the other hand, injected NPs can be quickly surrounded by pentameric IgM antibodies with a far bigger size than single-molecule memory IgG or complement molecules. The diversity of the corona changes the identity of the particles ruling their biological fate [125,126]. However, the bio-corona generated onto the NP surface, in turn, depends on NP physicochemical characteristics determining what is actually facing the cells. As a consequence, it mediates the uptake and/or activate different signaling pathways [127]. Many of the subset of serum molecules that interact with NPs, such as complement [128] and immunoglobulins, are immune-active, managing the interaction with immune cells in the different tissues.

The physicochemical properties, like NP surface charge, affect the nanomaterial biodistribution, though the current knowledge cannot enable general conclusions. NPs can distribute to various organs, and may retain the same original structure or not, being modified and metabolized [129]. It is worth distinguishing between acute and chronic exposure to NPs, since exposure time determines the optimal technique to monitor the interactions of NPs with the immune system. A high percentage of NPs can normally be sequestered in the liver, or in other organs, including spleen, lymph nodes, and bone marrow. Notwithstanding the importance of finding the threshold dose inducing toxicity, many experiments performed, *in vivo*, use amounts of metal NPs which are unlikely to be reasonable in human exposure through the considered routes [130]. The investigation of very low doses of NPs administered orally or by skin adsorption would be closer to the potential chronic exposure humans may have throughout their life using NP-loaded commercial products. The data obtained, in this way, help in understanding how a few particles can trigger immune reactions without showing massive toxicity or organ failure, “simply” due to the accumulation of metals and release of metal ions [131]. Furthermore, these organs are guarded by specialized macrophages, as part of the mononuclear phagocytes system (MPS) usually dealing with the uptake and metabolism of foreign molecules, which amplifies the retention and increases the ion release by ROS formation and lysosome-dependent metal NPs [132]. NP size is another critical parameter affecting final fate in the organism. For instance, NPs smaller than 5 nm in diameter are excreted in the urine, throughout the capillaries of the renal tubes [133], limiting their persistence into the body, otherwise micron-sized particles. Interestingly, NP size has been shown to influence the generation of CD8 or CD4 type I T cell responses [134,135], probably determined by the width of the NP surface in contact with cells, or the amount of absorbed active molecules reacting with membrane receptors. These hints guide us in the choice of the proper methods to reveal the mechanisms of NP-induced immune responses. Nonetheless, immune systems, hence, immune responses, display differences among animal species. The use of animal models in immunology is of indubitable importance. Differences and similarities to the human immune system of the experimental available animal models are still a matter of debate. As a representative example, one of the most used allergy models in mouse, namely ovalbumin as an allergen in BALB/c mice, does not reflect the development of an allergic reaction in humans [136].

**Figure 1:**
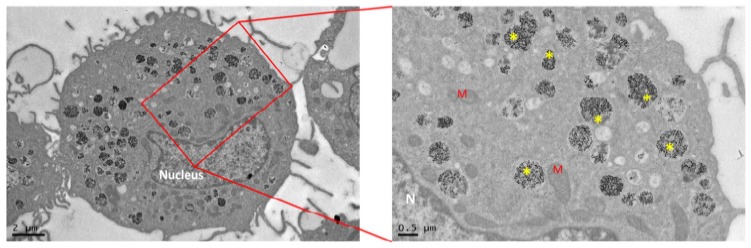
TEM images of human macrophage. Dark electron dense structures represent platinum nanoparticles within intracellular vesicles. M: mitochondria; N: Nucleus; *: NPs.

As well as for *in vitro* studies, there is a lack of standard controls for NP immunotoxicological studies [137]. While many types of nanomaterials have been linked to certain types of immunotoxicity, standardization of the methods for each material is very difficult. Usually, the immune response to nanomaterials, *in vivo*, relies on traditional tests, with special attention to the innate immune reactions. These include histochemical analysis of the tissue in the site of NP release (e.g., skin or muscle) to monitor of the recruitment of immune cell, as well as the local cell activation and presence of inflammatory mediators, and possible initiation of adaptive immunity in lymph nodes (LNs). Metallic NPs are relatively straightforward to identify by microscopy in cells and tissues, due to their opacity in visible light and electron density in transmission electron microscopy (TEM) (Figure 1) [88]. As previously stated, blood concentration of inflammatory mediators and immunoglobulin over a precise time period is usually performed depending on the experimental model and the administration route. As well as for *in vitro* analysis, specific standardized controls for NPs are missing, and conventional molecules are generally used, such as lipopolysaccharide (LPS) as a positive control for cytokine induction [121]. Although LPS induces reliable immune responses, mimicking the effects of the Gram-negative bacterial wall, it is debated whether this molecule is the appropriate control for metallic NPs. Indeed, the specific LPS cell receptors (e.g., CD14 and beta2 integrins) or the soluble LPS-binding protein LBP, trigger precise signaling cascades which are not tailored for artificial nanomaterials of any kind. Metal NPs, hence, do not stimulate the same pathways of LPS, hiding potential unexpected inflammatory responses.

## 4. Conclusions

The study of metallic nanomaterials effects on the immune system, their detection, and evaluation, requires cautious and meticulous characterization to avoid erroneous interpretations of the data. The main difficulty is related to the interference that material structures at the nanoscale can introduce into the standard methods currently used for chemicals or biological molecules. Although many studies have demonstrated that metallic NPs can have differential effects on the immune system and differential interaction within the leucocytes, the conclusions may vary using diverse experimental models, including different animal species. Future results will be achieved by a coordinated effort of chemists and immunologists to unravel nanomaterial–immune system interactions, and the appropriate investigation methodology.

## Figures and Tables

**Table 1:** Examples of frequently used metal nanoparticles in biomedicine.

Application	Nanoparticles	References
Antimicrobial	Ag	Hardman et al., Lansdown, Elechiguerra et al. [30,31,32]
	Cu	Cioffi et al. [40]
	TiO_2_	Kubacka et al. [39]
Cancer therapy	Ag	Wieder et al. [44]
	Au	Pissuwan et al., Zharov et al. [22,43]
Diagnostic	Au	Valentini et al. [25]
Drug delivery	Au	Voskerician et al. [24]
	Al	Tyner et al. [26]
Imaging	Ag	Caro et al. [11]
	Au	Sharma et al., Manohar et al., Cole et al., [9,20,21]
	Pt	Deyhimihaghighi et al. [19]
	Pd	Park et al. [12]
	Fe_2_O_3_	Corot et al., Gupta et al. [27,28]
	QDs	Wang et al., Kim et al., Stroh et al., Michalet et al. [14,15,16,17]
ROS scavenging	Pt	Moglianetti et al., Gatto et al. [45,46]
Wound healing	Ag	Poon et al., Fong et al. [41,42]

## References

[1] Moghimi S.M., Hunter A.C., Murray J.C. (2005). Nanomedicine: current status and future prospects. FASEB J..

[2] Riehemann K., Schneider S.W., Luger T.A., Godin B., Ferrari M., Fuchs H. (2009). Nanomedicine-Challenge and Perspectives. Angew. Chem. Int. Ed..

[3] Dreaden E.C., Alkilany A.M., Huang X., Murphy C.J., El-Sayed M.A. (2012). The golden age: gold nanoparticles for biomedicine. Chem. Soc. Rev..

[4] Jans H., Huo Q. (2012). Gold nanoparticle-enabled biological and chemical detection and analysis. Chem. Soc. Rev..

[5] Schütz C.A., Juillerat-Jeanneret L., Mueller H., Lynch I., Riediker M. (2013). Therapeutic nanoparticles in clinics and under clinical evaluation. Nanomedicine.

[6] De Jong W.H., Borm P.J.A. (2008). Drug delivery and nanoparticles: applications and hazards. Int. J. Nanomed..

[7] Schrand A.M., Rahman M.F., Hussain S.M., Schlager J.J., Smith D.A., Syed A.F. (2010). Metal-based nanoparticles and their toxicity assessment. Wiley Interdiscip. Rev. Nanomed. Nanobiotechnol..

[8] Liong M., Lu J., Kovochich M., Xia T., Ruehm S.G., Nel A.E., Tamanoi F., Zink J.I. (2008). Multifunctional inorganic nanoparticles for imaging, targeting, and drug delivery. ACS Nano.

[9] Sharma P., Brown S.C., Bengtsson N., Zhang Q., Walter G.A., Grobmyer S.R., Santra S., Jiang H., Scott E.W., Moudgil B.M. (2008). Gold-Speckled Multimodal Nanoparticles for Noninvasive Bioimaging. Chem. Mater..

[10] Skebo J.E., Grabinski C.M., Schrand A.M., Schlager J.J., Hussain S.M. (2007). Assessment of metal nanoparticle agglomeration, uptake, and interaction using high-illuminating system. Int. J. Toxicol..

[11] Caro C., Castilo P.M., Klippstein R., Pozo D., Zaderenko A.P. (2010). Silver nanoparticles: Sensing and imaging applications. Silver Nanoparticles.

[12] Park J.-B., Lee J.H., Choi H.-R. (2007). Three-dimensional imaging of stacked Pd nanoparticles by electron tomography. Appl. Phys. Lett..

[13] Pedone D., Moglianetti M., De Luca E., Bardi G., Pompa P.P. (2017). Platinum nanoparticles in nanobiomedicine. Chem. Soc. Rev..

[14] Wang F., Tan W.B., Zhang Y., Fan X., Wang M. (2006). Luminescent nanomaterials for biological labelling. Nanotechnology.

[15] Kim S., Lim Y.T., Soltesz E.G., De Grand A.M., Lee J., Nakayama A., Parker J.A., Mihaljevic T., Laurence R.G., Dor D.M. (2004). Near-infrared fluorescent type II quantum dots for sentinel lymph node mapping. Nat. Biotechnol..

[16] Stroh M., Zimmer J.P., Duda D.G., Levchenko T.S., Cohen K.S., Brown E.B., Scadden D.T., Torchilin V.P., Bawendi M.G., Fukumura D. (2005). Quantum dots spectrally distinguish multiple species within the tumor milieu *in vivo*. Nat. Med..

[17] Michalet X., Pinaud F.F., Bentolila L.A., Tsay J.M., Doose S., Li J.J., Sundaresan G., Wu A.M., Gambhir S.S., Weiss S. (2005). Quantum dots for live cells, *in vivo* imaging, and diagnostics. Science.

[18] Shan X., Díez-Pérez I., Wang L., Wiktor P., Gu Y., Zhang L., Wang W., Lu J., Wang S., Gong Q. (2012). Imaging the electrocatalytic activity of single nanoparticles. Nat. Nanotechnol..

[19] Deyhimihaghighi N., Noor N.M., Soltani N., Jorfi R., Erfani Haghir M., Adenan M.Z., Saion E., Khandaker M.U. (2014). Contrast enhancement of magnetic resonance imaging (MRI) of polymer gel dosimeter by adding Platinum nano-particles. J. Phys. Conf. Ser..

[20] Manohar N., Reynoso F.J., Diagaradjane P., Krishnan S., Cho S.H. (2016). Quantitative imaging of gold nanoparticle distribution in a tumor-bearing mouse using benchtop x-ray fluorescence computed tomography. Sci. Rep..

[21] Cole L.E., Ross R.D., Tilley J.M., Vargo-Gogola T., Roeder R.K. (2015). Gold nanoparticles as contrast agents in x-ray imaging and computed tomography. Nanomedicine.

[22] Pissuwan D., Valenzuela S.M., Killingsworth M.C., Xu X., Cortie M.B. (2007). Targeted destruction of murine macrophage cells with bioconjugated gold nanorods. J. Nanoparticle Res..

[23] Yi H., Leunissen J.L.M., Shi G.-M., Gutekunst C.-A., Hersch S.M. (2001). A novel procedure for pre-embedding double immunogold–silver labeling at the ultrastructural level. J. Histochem. Cytochem..

[24] Voskerician G., Shive M.S., Shawgo R.S., von Recum H., Anderson J.M., Cima M.J., Langer R. (2003). Biocompatibility and biofouling of MEMS drug delivery devices. Biomaterials.

[25] Valentini P., Galimberti A., Mezzasalma V., De Mattia F., Casiraghi M., Labra M., Pompa P.P. (2017). DNA barcoding meets nanotechnology: Development of a universal colorimetric test for food authentication. Angew. Chem. Int. Ed..

[26] Tyner K.M., Schiffman S.R., Giannelis E.P. (2004). Nanobiohybrids as delivery vehicles for camptothecin. J. Control. Release.

[27] Corot C., Robert P., Idée J.-M., Port M. (2006). Recent advances in iron oxide nanocrystal technology for medical imaging. Adv. Drug Deliv. Rev..

[28] Gupta A.K., Gupta M. (2005). Synthesis and surface engineering of iron oxide nanoparticles for biomedical applications. Biomaterials.

[29] Davenport K., Keeley F.X. (2005). Evidence for the use of silver-alloy-coated urethral catheters. J. Hosp. Infect..

[30] Hardman S., Cope A., Swann A., Bell P.R.F., Naylor A.R., Hayes P.D. (2004). An *in vitro* model to compare the antimicrobial activity of silver-coated versus rifampicin-soaked vascular grafts. Ann. Vasc. Surg..

[31] Lansdown A.B.G. (2006). Silver in health care: antimicrobial effects and safety in use. Curr. Probl. Dermatol..

[32] Elechiguerra J., Burt J.L., Morones J.R., Camacho-Bragado A., Gao X., Lara H.H., Yacaman M. (2005). Interaction of silver nanoparticles with HIV-1. J. Nanobiotechnology.

[33] Alt V., Bechert T., Steinrücke P., Wagener M., Seidel P., Dingeldein E., Domann E., Schnettler R. (2004). An *in vitro* assessment of the antibacterial properties and cytotoxicity of nanoparticulate silver bone cement. Biomaterials.

[34] Morones J.R., Elechiguerra J.L., Camacho A., Holt K., Kouri J.B., Ramírez J.T., Yacaman M.J. (2005). The bactericidal effect of silver nanoparticles. Nanotechnology.

[35] Percival S.L., Bowler P.G., Dolman J. (2007). Antimicrobial activity of silver-containing dressings on wound microorganisms using an *in vitro* biofilm model. Int. Wound J..

[36] Samuel U., Guggenbichler J. (2004). Prevention of catheter-related infections: the potential of a new nano-silver impregnated catheter. Int. J. Antimicrob. Agents.

[37] Vigneshwaran N., Kathe A.A., Varadarajan P.V., Nachane R.P., Balasubramanya R.H. (2007). Functional finishing of cotton fabrics using silver nanoparticles. J. Nanosci. Nanotechnol..

[38] Jain J., Arora S., Rajwade J.M., Omray P., Khandelwal S., Paknikar K.M. (2009). Silver nanoparticles in therapeutics: Development of an antimicrobial gel formulation for topical use. Mol. Pharm..

[39] Kubacka A., Diez M.S., Rojo D., Bargiela R., Ciordia S., Zapico I., Albar J.P., Barbas C., Martins dos Santos V.A.P., Fernández-García M. (2015). Understanding the antimicrobial mechanism of TiO2-based nanocomposite films in a pathogenic bacterium. Sci. Rep..

[40] Cioffi N., Ditaranto N., Torsi L., Picca R.A., De Giglio E., Sabbatini L., Novello L., Tantillo G., Bleve-Zacheo T., Zambonin P.G. (2005). Synthesis, analytical characterization and bioactivity of Ag and Cu nanoparticles embedded in poly-vinyl-methyl-ketone films. Anal. Bioanal. Chem..

[41] Poon V.K.M., Burd A. (2004). *In vitro* cytotoxity of silver: implication for clinical wound care. Burns.

[42] Fong J., Wood F. (2006). Nanocrystalline silver dressings in wound management: A review. Int. J. Nanomed..

[43] Zharov V.P., Galitovskaya E.N., Johnson C., Kelly T. (2005). Synergistic enhancement of selective nanophotothermolysis with gold nanoclusters: Potential for cancer therapy. Lasers Surg. Med..

[44] Wieder M.E., Hone D.C., Cook M.J., Handsley M.M., Gavrilovic J., Russell D.A. (2006). Intracellular photodynamic therapy with photosensitizer-nanoparticle conjugates: cancer therapy using a ‘Trojan horse’. Photochem. Photobiol. Sci..

[45] Moglianetti M., De Luca E., Pedone D., Marotta R., Catelani T., Sartori B., Amenitsch H., Retta S.F., Pompa P.P. (2016). Platinum nanozymes recover cellular ROS homeostasis in an oxidative stress-mediated disease model. Nanoscale.

[46] Gatto F., Moglianetti M., Pompa P., Bardi G. (2018). Platinum nanoparticles decrease reactive oxygen species and modulate gene expression without alteration of immune responses in THP-1 Monocytes. Nanomaterials.

[47] Nel A., Xia T., Mädler L., Li N. (2006). Toxic potential of materials at the nanolevel. Science.

[48] Fadeel B., Garcia-Bennett A.E. (2010). Better safe than sorry: Understanding the toxicological properties of inorganic nanoparticles manufactured for biomedical applications. Adv. Drug Deliv. Rev..

[49] Krpetić Z., Saleemi S., Prior I.A., Sée V., Qureshi R., Brust M. (2011). Negotiation of intracellular membrane barriers by TAT-modified gold nanoparticles. ACS Nano.

[50] Farrera C., Fadeel B. (2015). It takes two to tango: Understanding the interactions between engineered nanomaterials and the immune system. Eur. J. Pharm. Biopharm..

[51] Rasmussen K., Rauscher H., Mech A., Riego Sintes J., Gilliland D., González M., Kearns P., Moss K., Visser M., Groenewold M. (2018). Physico-chemical properties of manufactured nanomaterials—Characterisation and relevant methods. An outlook based on the OECD Testing Programme. Regul. Toxicol. Pharmacol..

[52] Najafi-Hajivar S., Zakeri-Milani P., Mohammadi H., Niazi M., Soleymani-Goloujeh M., Baradaran B., Valizadeh H. (2016). Overview on experimental models of interactions between nanoparticles and the immune system. Biomed. Pharmacother..

[53] Abbas A.K., Lichtman A.H., Pillai S. (2015). Cellular and Molecular Immunology.

[54] Martin M., Blom A.M. (2016). Complement in removal of the dead—balancing inflammation. Immunol. Rev..

[55] Ricklin D., Hajishengallis G., Yang K., Lambris J.D. (2010). Complement: A key system for immune surveillance and homeostasis. Nat. Immunol..

[56] Tavano R., Gabrielli L., Lubian E., Fedeli C., Visentin S., Polverino De Laureto P., Arrigoni G., Geffner-Smith A., Chen F., Simberg D. (2018). C1q-mediated complement activation and C3 opsonization trigger recognition of stealth poly(2-methyl-2-oxazoline)-coated silica nanoparticles by human phagocytes. ACS Nano.

[57] Silva A.L., Peres C., Conniot J., Matos A.I., Moura L., Carreira B., Sainz V., Scomparin A., Satchi-Fainaro R., Préat V. (2017). Nanoparticle impact on innate immune cell pattern-recognition receptors and inflammasomes activation. Semin. Immunol..

[58] Fadeel B. (2012). Clear and present danger? Engineered nanoparticles and the immune system. Swiss Med. Wkly..

[59] Klippstein R., Fernandez-Montesinos R., Pozo D., Zaderenko A.P., Pozo D. (2010). Silver nanoparticles interactions with the immune system: Implications for health and disease. Silver Nanoparticles.

[60] Nakayama M. (2018). Macrophage recognition of crystals and nanoparticles. Front. Immunol..

[61] Martinon F., Burns K., Tschopp J. (2002). The inflammasome: A molecular platform triggering activation of inflammatory caspases and processing of proIL-beta. Mol. Cell.

[62] Rathinam V.A.K., Fitzgerald K.A. (2016). Inflammasome complexes: Emerging mechanisms and effector functions. Cell.

[63] Sakhtianchi R., Minchin R.F., Lee K.-B., Alkilany A.M., Serpooshan V., Mahmoudi M. (2013). Exocytosis of nanoparticles from cells: Role in cellular retention and toxicity. Adv. Colloid Interface Sci..

[64] Fröhlich E. (2016). Cellular elimination of nanoparticles. Environ. Toxicol. Pharmacol..

[65] Albanese A., Tang P.S., Chan W.C.W. (2012). The effect of nanoparticle size, shape, and surface chemistry on biological systems. Annu. Rev. Biomed. Eng..

[66] Gamucci O., Bertero A., Gagliardi M., Bardi G. (2014). Biomedical nanoparticles: Overview of their surface immune-compatibility. Coatings.

[67] Kroll A., Pillukat M.H., Hahn D., Schnekenburger J. (2009). Current *in vitro* methods in nanoparticle risk assessment: Limitations and challenges. Eur. J. Pharm. Biopharm..

[68] Park M.V., Lankveld D.P., van Loveren H., de Jong W.H. (2009). The status of *in vitro* toxicity studies in the risk assessment o nanomaterials. Nanomedicine.

[69] Wick P., Grafmueller S., Petri-Fink A., Rothen-Rutishauser B. (2014). Advanced human *in vitro* models to assess metal oxide nanoparticle-cell interactions. MRS Bull..

[70] Kämpfer A.A.M., Urbán P., Gioria S., Kanase N., Stone V., Kinsner-Ovaskainen A. (2017). Development of an *in vitro* co-culture model to mimic the human intestine in healthy and diseased state. Toxicol. In Vitro..

[71] Dobrovolskaia M.A., Shurin M., Shvedova A.A. (2016). Current understanding of interactions between nanoparticles and the immune system. Toxicol. Appl. Pharmacol..

[72] Petrarca C., Clemente E., Amato V., Pedata P., Sabbioni E., Bernardini G., Iavicoli I., Cortese S., Niu Q., Otsuki T. (2015). Engineered metal based nanoparticles and innate immunity. Clin. Mol. Allergy.

[73] Leso V., Fontana L., Mauriello M., Iavicoli I. (2016). Occupational risk assessment of engineered nanomaterials: limits, challenges and opportunities. Curr. Nanosci..

[74] Savolainen K., Alenius H., Norppa H., Pylkkänen L., Tuomi T., Kasper G. (2010). Risk assessment of engineered nanomaterials and nanotechnologies—A review. Toxicology.

[75] Braun G.B., Friman T., Pang H.-B., Pallaoro A., Hurtado de Mendoza T., Willmore A.-M.A., Kotamraju V.R., Mann A.P., She Z.-G., Sugahara K.N. (2014). Etchable plasmonic nanoparticle probes to image and quantify cellular internalization. Nat. Mater..

[76] Ong K.J., MacCormack T.J., Clark R.J., Ede J.D., Ortega V.A., Felix L.C., Dang M.K.M., Ma G., Fenniri H., Veinot J.G.C. (2014). Widespread nanoparticle-assay interference: Implications for nanotoxicity testing. PLoS ONE.

[77] Veranth J.M., Kaser E.G., Veranth M.M., Koch M., Yost G.S. (2007). Cytokine responses of human lung cells (BEAS-2B) treated with micron-sized and nanoparticles of metal oxides compared to soil dusts. Part. Fibre Toxicol..

[78] Granchi D., Ciapetti G., Savarino L., Cavedagna D., Donati M.E., Pizzoferrato A. (1996). Assessment of metal extract toxicity on human lymphocytes cultured *in vitro*. J. Biomed. Mater. Res..

[79] Davis R.R., Lockwood P.E., Hobbs D.T., Messer R.L.W., Price R.J., Lewis J.B., Wataha J.C. (2007). *In vitro* biological effects of sodium titanate materials. J. Biomed. Mater. Res. Part B Appl. Biomater..

[80] Dulkeith E., Ringler M., Klar T.A., Feldmann J., Munoz Javier A., Parak W.J. (2005). Gold nanoparticles quench fluorescence by phase induced radiative rate suppression. Nano Lett..

[81] Kawabata T.T., Evans E.W. (2012). Development of immunotoxicity testing strategies for immunomodulatory drugs. Toxicol. Pathol..

[82] Mosmann T. (1983). Rapid colorimetric assay for cellular growth and survival: application to proliferation and cytotoxicity assays. J. Immunol. Methods.

[83] Chan F.K.-M., Moriwaki K., De Rosa M.J., Snow A., Lenardo M. (2013). Detection of necrosis by release of lactate dehydrogenase activity. Immune Homeostasis. Methods in Molecular Biology (Methods and Protocols).

[84] Vermes I., Haanen C., Reutelingsperger C. (2000). Flow cytometry of apoptotic cell death. J. Immunol. Methods.

[85] Aubry J.P., Blaecke A., Lecoanet-Henchoz S., Jeannin P., Herbault N., Caron G., Moine V., Bonnefoy J.Y. (1999). Annexin V used for measuring apoptosis in the early events of cellular cytotoxicity. Cytometry.

[86] Waterhouse N.J., Green D.R. (1999). Mitochondria and apoptosis: HQ or high-security prison?. J. Clin. Immunol..

[87] Borenfreund E., Puerner J.A. (1985). Toxicity determined *in vitro* by morphological alterations and neutral red absorption. Toxicol. Lett..

[88] Gatto F., Cagliani R., Catelani T., Guarnieri D., Moglianetti M., Pompa P.P., Bardi G. (2017). PMA-induced THP-1 macrophage differentiation is not impaired by citrate-coated platinum nanoparticles. Nanomaterials.

[89] Boraschi D., Costantino L., Italiani P. (2012). Interaction of nanoparticles with immunocompetent cells: nanosafety considerations. Nanomedicine.

[90] Guo L., Von Dem Bussche A., Buechner M., Yan A., Kane A.B., Hurt R.H. (2008). Adsorption of essential micronutrients by carbon nanotubes and the implications for nanotoxicity testing. Small.

[91] Shukla S., Priscilla A., Banerjee M., Bhonde R.R., Ghatak J., Satyam P.V., Sastry M. (2005). Porous gold nanospheres by controlled transmetalation reaction: A novel material for application in cell imaging. Chem. Mater..

[92] Wörle-Knirsch J.M., Pulskamp K., Krug H.F. (2006). Oops they did it again! Carbon nanotubes hoax scientists in viability assays. Nano Lett..

[93] Pulskamp K., Diabaté S., Krug H.F. (2007). Carbon nanotubes show no sign of acute toxicity but induce intracellular reactive oxygen species in dependence on contaminants. Toxicol. Lett..

[94] Suska F., Gretzer C., Esposito M., Tengvall P., Thomsen P. (2005). Monocyte viability on titanium and copper coated titanium. Biomaterials.

[95] Segal M.S., Beem E. (2001). Effect of pH, ionic charge, and osmolality on cytochrome *c* -mediated caspase-3 activity. Am. J. Physiol. Physiol..

[96] Stennicke H.R., Salvesen G.S. (1997). Biochemical characteristics of caspases-3, -6, -7, and -8. J. Biol. Chem..

[97] Meulenkamp E.A. (1998). Size dependence of the dissolution of ZnO nanoparticles. J. Phys. Chem. B.

[98] Isakovic A., Markovic Z., Todorovic-Markovic B., Nikolic N., Vranjes-Djuric S., Mirkovic M., Dramicanin M., Harhaji L., Raicevic N., Nikolic Z. (2006). Distinct cytotoxic mechanisms of pristine versus hydroxylated fullerene. Toxicol. Sci..

[99] Fubini B., Hubbard A. (2003). Reactive oxygen species (ROS) and reactive nitrogen species (RNS) generation by silica in inflammation and fibrosis. Free Radic. Biol. Med..

[100] Hasnat M.A., Uddin M.M., Samed A.J.F., Alam S.S., Hossain S. (2007). Adsorption and photocatalytic decolorization of a synthetic dye erythrosine on anatase TiO2 and ZnO surfaces. J. Hazard. Mater..

[101] Schafer F.Q., Qian S.Y., Buettner G.R. (2000). Iron and free radical oxidations in cell membranes. Cell. Mol. Biol. (Noisy-le-grand).

[102] Dobson J. (2001). Nanoscale biogenic iron oxides and neurodegenerative disease. FEBS Lett..

[103] Aranda A., Sequedo L., Tolosa L., Quintas G., Burello E., Castell J.V., Gombau L. (2013). Dichloro-dihydro-fluorescein diacetate (DCFH-DA) assay: A quantitative method for oxidative stress assessment of nanoparticle-treated cells. Toxicol. In Vitro.

[104] Rahman I., Kode A., Biswas S.K. (2007). Assay for quantitative determination of glutathione and glutathione disulfide levels using enzymatic recycling method. Nat. Protoc..

[105] Kroll A., Pillukat M.H., Hahn D., Schnekenburger J. (2012). Interference of engineered nanoparticles with *in vitro* toxicity assays. Arch. Toxicol..

[106] Royall J.A., Ischiropoulos H. (1993). Evaluation of 2′,7′-Dichlorofluorescin and Dihydrorhodamine 123 as Fluorescent Probes for Intracellular H_2_O_2_ in Cultured Endothelial Cells. Arch. Biochem. Biophys..

[107] Smaili S.S., Pereira G.J.S., Costa M.M., Rocha K.K., Rodrigues L., do Carmo L.G., Hirata H., Hsu Y.-T. (2013). The role of calcium stores in apoptosis and autophagy. Curr. Mol. Med..

[108] Gamucci O., Bardi G., Olimpia G. (2015). Cerium dioxide nanoparticles selectively up - regulate C - C chemokine receptor 2 and CD16 expression on human monocytes. EURO-NanoTox-Letters.

[109] Gatto F., Troncoso O.P., Brunetti V., Malvindi M.A., Pompa P.P., Torres F.G., Bardi G. (2016). Human monocyte response to Andean-native starch nanoparticles. Starch Stärke.

[110] Bertero A., Boni A., Gemmi M., Gagliardi M., Bifone A., Bardi G. (2014). Surface functionalisation regulates polyamidoamine dendrimer toxicity on blood–brain barrier cells and the modulation of key inflammatory receptors on microglia. Nanotoxicology.

[111] Shapiro H.M. (2003). Practical Flow Cytometry.

[112] Matsumoto B. (2002). Cell Biological Applications of Confocal Microscopy.

[113] Baggiolini M. (1998). Chemokines and leukocyte traffic. Nature.

[114] Justus C.R., Leffler N., Ruiz-Echevarria M., Yang L.V. (2014). *In vitro* cell migration and invasion assays. J. Vis. Exp..

[115] Schöler N., Olbrich C., Tabatt K., Müller R.H., Hahn H., Liesenfeld O. (2001). Surfactant, but not the size of solid lipid nanoparticles (SLN) influences viability and cytokine production of macrophages. Int. J. Pharm..

[116] Vallhov H., Qin J., Johansson S.M., Ahlborg N., Muhammed M.A., Scheynius A., Gabrielsson S. (2006). The Importance of an Endotoxin-Free Environment during the Production of Nanoparticles Used in Medical Applications. Nano Lett..

[117] Li Y., Fujita M., Boraschi D. (2017). Endotoxin contamination in nanomaterials leads to the misinterpretation of immunosafety results. Front. Immunol..

[118] Dobrovolskaia M.A., Patri A.K., Potter T.M., Rodriguez J.C., Hall J.B., McNeil S.E. (2012). Dendrimer-induced leukocyte procoagulant activity depends on particle size and surface charge. Nanomedicine.

[119] Inoue K., Takano H., Yanagisawa R., Hirano S., Kobayashi T., Fujitani Y., Shimada A., Yoshikawa T. (2007). Effects of inhaled nanoparticles on acute lung injury induced by lipopolysaccharide in mice. Toxicology.

[120] Inoue K.-I., Takano H., Yanagisawa R., Hirano S., Sakurai M., Shimada A., Yoshikawa T. (2006). Effects of airway exposure to nanoparticles on lung inflammation induced by bacterial endotoxin in mice. Environ. Health Perspect..

[121] Dobrovolskaia M.A. (2015). Pre-clinical immunotoxicity studies of nanotechnology-formulated drugs: Challenges, considerations and strategy. J. Control. Release.

[122] Fischer H.C., Chan W.C. (2007). Nanotoxicity: The growing need for *in vivo* study. Curr. Opin. Biotechnol..

[123] Oberdörster G., Maynard A., Donaldson K., Castranova V., Fitzpatrick J., Ausman K., Carter J., Karn B., Kreyling W., Lai D. (2005). Principles for characterizing the potential human health effects from exposure to nanomaterials: elements of a screening strategy. Part. Fibre Toxicol..

[124] Jenkin C.R., Rowley D. (1961). The role of opsonins in the clearance of living and inert particles by cells of the reticuloendothelial system. J. Exp. Med..

[125] Neagu M., Piperigkou Z., Karamanou K., Engin A.B., Docea A.O., Constantin C., Negrei C., Nikitovic D., Tsatsakis A. (2017). Protein bio-corona: critical issue in immune nanotoxicology. Arch. Toxicol..

[126] Treuel L., Docter D., Maskos M., Stauber R.H. (2015). Protein corona—from molecular adsorption to physiological complexity. Beilstein J. Nanotechnol..

[127] Cedervall T., Lynch I., Lindman S., Berggård T., Thulin E., Nilsson H., Dawson K.A., Linse S. (2007). Understanding the nanoparticle-protein corona using methods to quantify exchange rates and affinities of proteins for nanoparticles. Proc. Natl. Acad. Sci. USA.

[128] Reddy S.T., van der Vlies A.J., Simeoni E., Angeli V., Randolph G.J., O’Neil C.P., Lee L.K., Swartz M.A., Hubbell J.A. (2007). Exploiting lymphatic transport and complement activation in nanoparticle vaccines. Nat. Biotechnol..

[129] Borm P., Klaessig F.C., Landry T.D., Moudgil B., Pauluhn J., Thomas K., Trottier R., Wood S. (2006). Research strategies for safety evaluation of nanomaterials, Part V: Role of dissolution in biological fate and effects of nanoscale particles. Toxicol. Sci..

[130] Fu Y., Zhang Y., Chang X., Zhang Y., Ma S., Sui J., Yin L., Pu Y., Liang G. (2014). Systemic immune effects of titanium dioxide nanoparticles after repeated intratracheal instillation in rat. Int. J. Mol. Sci..

[131] Hong J., Wang L., Zhao X., Yu X., Sheng L., Xu B., Liu D., Zhu Y., Long Y., Hong F. (2014). Th2 factors may be involved in TiO_2_ NP-induced hepatic inflammation. J. Agric. Food Chem..

[132] Saba T.M. (1970). Physiology and physiopathology of the reticuloendothelial system. Arch. Intern. Med..

[133] Soo Choi H., Liu W., Misra P., Tanaka E., Zimmer J.P., Itty Ipe B., Bawendi M.G., Frangioni J.V. (2007). Renal clearance of quantum dots. Nat. Biotechnol..

[134] Fifis T., Gamvrellis A., Crimeen-Irwin B., Pietersz G.A., Li J., Mottram P.L., McKenzie I.F.C., Plebanski M. (2004). Size-dependent immunogenicity: therapeutic and protective properties of nano-vaccines against tumors. J. Immunol..

[135] Gamvrellis A., Leong D., Hanley J.C., Xiang S.D., Mottram P., Plebanski M. (2004). Vaccines that facilitate antigen entry into dendritic cells. Immunol. Cell Biol..

[136] Chen C., Sun N., Li Y., Jia X. (2013). A BALB/c mouse model for assessing the potential allergenicity of proteins: Comparison of allergen dose, sensitization frequency, timepoint and sex. Food Chem. Toxicol..

[137] Dobrovolskaia M.A., Germolec D.R., Weaver J.L. (2009). Evaluation of nanoparticle immunotoxicity. Nat. Nanotechnol..

